# Proteomic identification of resistin as a NET-associated neutrophil activity marker in sepsis

**DOI:** 10.1186/s40560-026-00954-8

**Published:** 2026-10-13

**Authors:** Ema Hashiguchi, Akane Shinkai, Michiko Katsuno, Mineji Hayakawa, Takaaki Totoki, Masayoshi Tasaki, Takashi Ito

**Affiliations:** 1 Graduate School of Health Sciences, Kumamoto University, Kumamoto, Japan; 2 Department of Hematology and Immunology, Faculty of Life Sciences, Kumamoto University, 4-24-1 Kuhonji, Kumamoto, 862-0976 Japan; 3 Department of Emergency and General Medicine, Sapporo City General Hospital, Sapporo, Japan; 4 Department of Emergency and Critical Care Medicine, Osaka Medical and Pharmaceutical University, Takatsuki, Japan; 5 Department of Anesthesiology and Critical Care Medicine, Kyushu University, Fukuoka, Japan; 6 Department of Clinical Biosciences, Faculty of Life Sciences, Kumamoto University, Kumamoto, Japan

**Keywords:** Sepsis, Neutrophil extracellular traps (NETs), Immunothrombosis, Organ failure

## Abstract

**Background:**

Neutrophil extracellular traps (NETs) contribute to organ dysfunction in sepsis, yet clinically accessible biomarkers reflecting NET-associated pathology remain limited. We sought to identify circulating proteins associated with NET formation and evaluate their clinical relevance in sepsis.

**Methods:**

Unbiased proteomic profiling of stimulated human neutrophil supernatants and serum from murine sepsis was performed to identify proteins released during NET formation and elevated in vivo. Candidate biomarkers were selected through integration of these datasets and filtering based on cell type-specific expression profiles, followed by validation in patients with sepsis and healthy controls.

**Results:**

Integrative analysis identified five candidate circulating markers associated with NET formation in sepsis: cathelicidin antimicrobial peptide, lactotransferrin, neutrophil gelatinase-associated lipocalin (NGAL), resistin, and histone H4. Among these, resistin, a recently recognized neutrophil-associated protein, and NGAL demonstrated the strongest associations with sepsis-associated complications. Both markers were elevated in patients with sepsis and correlated with circulating cell-free DNA levels. In receiver operating characteristic curve analyses, resistin discriminated multiple organ failure (AUC, 0.83) and disseminated intravascular coagulation (AUC, 0.81), whereas NGAL demonstrated similarly robust performance (AUC, 0.84 and 0.79, respectively). Both markers outperformed conventional clinical parameters for discriminating sepsis-associated complications.

**Conclusions:**

These findings identify resistin as a circulating marker associated with NET formation that captures key features of sepsis-associated immunothrombosis and organ failure and may improve assessment of disease severity and risk stratification.

**Supplementary Information:**

The online version contains supplementary material available at https://doi.org/10.1186/s40560-026-00954-8.

## Background

Sepsis is a life-threatening organ dysfunction resulting from a dysregulated host response to infection [1]. According to the World Health Organization, there were an estimated 48.9 million cases of sepsis and 11 million sepsis-related deaths worldwide in 2020, accounting for approximately 20% of all global deaths [2, 3]. In addition to its substantial mortality burden, sepsis imposes considerable economic costs; in high-income countries, the healthcare expenditure per patient is estimated to be approximately USD 32,000 [4]. Despite its global impact, the underlying pathophysiological mechanisms of sepsis remain unclear.

Neutrophils play a central role in host defense against infection but are also implicated in the development of organ dysfunction [5]. These cells exert antimicrobial effects through multiple mechanisms, including chemotaxis, phagocytosis, and enzyme- and reactive oxygen species (ROS)-mediated intracellular killing. Recently, the formation of neutrophil extracellular traps (NETs) has been recognized as an additional effector function of neutrophils [6, 7]. NETs consist of a web-like structure of extracellular DNA decorated with antimicrobial components, such as myeloperoxidase (MPO), neutrophil elastase (NE), and histones. Through this structure, NETs can entrap and immobilize pathogens, thereby limiting their dissemination [7–9].

In addition to their protective roles, accumulating evidence suggests that NETs contribute to the pathogenesis of organ dysfunction in sepsis [10]. Histones released into the extracellular milieu during NET formation are highly cationic and can interact with cellular membranes, leading to pore formation and cytotoxicity [11]. Circulating histones have also been shown to directly injure endothelial cells and disrupt their anticoagulant properties [12]. Furthermore, extracellular DNA, another key component of NETs, activates coagulation factors XII and XI, thereby promoting the intrinsic coagulation pathway and enhancing thrombogenesis [13]. In septic conditions, such NET-mediated prothrombotic activity has been linked to the development of disseminated intravascular coagulation (DIC), which contributes to organ failure and increased mortality [14].

Collectively, these findings highlight the critical role of NETs in the progression of organ dysfunction in sepsis. Accordingly, the detection of NETs in patient samples may provide valuable insights into disease mechanisms, enable the assessment of disease severity, and support therapeutic decision-making. However, NETs are predominantly released by neutrophils adhering to tissues or the vascular endothelium, and their detection currently relies largely on histopathological analysis of tissue specimens. This limitation hinders rapid and practical evaluation in clinical settings, particularly in acute conditions such as sepsis [15].

To address this challenge, the present study aimed to identify novel soluble biomarkers associated with NET formation that are detectable in serum or plasma. We conducted an unbiased proteomic screening of candidate molecules using both in vitro and in vivo samples, followed by validation using clinical samples.

## Methods

### Blood collection from healthy volunteers and isolation of blood cell fractions

All experiments involving human participants were conducted in accordance with the Declaration of Helsinki and performed under the study protocol approved by the Ethics Committee of Kumamoto University (Approval No. 2737 and 3245). Peripheral blood was collected from the median cubital vein of healthy volunteers after obtaining informed consent. A portion of blood was used for serum preparation and stored at −80 °C for subsequent enzyme-linked immunosorbent assay (ELISA). The remaining blood was anticoagulated with either EDTA or sodium citrate and used for the isolation of blood cell fractions. Neutrophils, monocytes, and lymphocytes were isolated using the EasySep™ Direct Human Neutrophil Isolation Kit, Monocyte Isolation Kit, and Total Lymphocyte Isolation Kit, respectively, according to the manufacturer’s instructions (StemCell Technologies, Vancouver, BC, Canada). For the isolation of platelets and erythrocytes, leukocytes were first removed using a White Blood Cell Syringe Filter (Pall, Port Washington, NY, USA). The filtered blood was centrifuged at 120×*g* for 10 min, resulting in separation into a platelet-rich supernatant and an erythrocyte-rich pellet. Whole blood and isolated neutrophil samples were then incubated with phycoerythrin (PE)-conjugated anti-human CD66b antibodies (BioLegend, San Diego, CA, USA), and analyzed using an RF-500 flow cytometer (Sysmex, Kobe, Japan).

### Neutrophil stimulation and collection of culture supernatants

Neutrophils (3 × 10^5^ cells/well) in RPMI medium (Nacalai Tesque, Kyoto, Japan) supplemented with 2% human albumin (Lee Biosolutions Inc., Maryland Heights, MO, USA) were seeded onto 4-well chamber slides (Thermo Fisher Scientific) and allowed to adhere for 1 h at 37 °C. The cells were then incubated in Opti-MEM (Gibco, Waltham, MA, USA) for an additional 10 min. For stimulation, phorbol 12-myristate 13-acetate (PMA; Promega, Madison, WI, USA) was added at a final concentration of 200 nM. After incubation for 4 h at 37 °C, the culture supernatants were collected and stored at − 80 °C until analysis. The cells were then fixed with 4% paraformaldehyde (Nacalai Tesque) and subjected to immunostaining.

### Immunofluorescence staining

Cells were permeabilized with Tris-buffered saline (Nacalai Tesque) containing 0.5% Triton X-100 (Sigma-Aldrich) for 1 min at room temperature, followed by blocking for 1 h at room temperature. Cells were incubated overnight at 4 °C with the following primary antibodies under gentle agitation: rabbit anti-MPO antibody (1:100; Calbiochem, Darmstadt, Germany), rabbit anti-NE antibody (1:100; Calbiochem), and mouse anti-resistin antibody (1:100; Abcam, Cambridge, UK). After washing, the cells were incubated for 1 h at room temperature with Alexa Fluor 488-conjugated secondary antibodies (1:100; Cell Signaling Technology, Danvers, MA, USA). Nuclear DNA was counterstained with 4′,6-diamidino-2-phenylindole (DAPI; Dojindo Laboratories, Kumamoto, Japan). Fluorescence images were acquired using an all-in-one fluorescence microscope (BZ-X1000; Keyence, Osaka, Japan).

### Serum collection from a mouse model of sepsis

Animal experiments were approved by the Animal Care and Use Committee of Kyushu University (Approval No. A22-393-0) and conducted in accordance with the institutional guidelines. Male C57BL/6J mice (Kyudo, Fukuoka, Japan) were anesthetized with sevoflurane, and sepsis was induced by cecal ligation and puncture (CLP) with minor modifications to a previously described method [16]. At 12 h after CLP or sham surgery, blood was collected from the inferior vena cava under anesthesia.

### Proteomic analysis

Neutrophil culture supernatants were depleted of albumin using Proteome Purify 2 (R&D Systems, Minneapolis, MN, USA). Serum samples from CLP and sham-operated mice were depleted of albumin, IgG, and transferrin using the Multiple Affinity Removal Spin Cartridge Mouse 3 (Agilent Technologies, Santa Clara, CA, USA). Protein concentrations were determined using the Pierce™ BCA Protein Assay Kit (Thermo Fisher Scientific). Isolated neutrophils, monocytes, lymphocytes, erythrocytes, and platelets were lysed using the lysis solution included in the EasyPep™ MS Sample Prep Kit (Thermo Fisher Scientific). For proteomic sample preparation, 50–300 µg of protein was processed using the EasyPep™ MS Sample Prep Kit according to the manufacturer’s instructions. Briefly, proteins were reduced and alkylated by carbamidomethylation of cysteine residues, followed by digestion with a Trypsin/Lys-C protease mixture and peptide cleanup procedure. The resulting peptides were dried and reconstituted in 100 µL of mass spectrometry-grade water containing 2% acetonitrile and 0.1% trifluoroacetic acid.

Liquid chromatography–tandem mass spectrometry (LC–MS/MS) analysis was performed using a Q-Exactive Orbitrap mass spectrometer. The peptides were loaded onto a trap column (PepMap Neo C18; 300 μm × 5 mm, 5 μm; Thermo Fisher Scientific) and subsequently separated on a nano-HPLC capillary column (75 μm × 125 mm, 3 μm; Nikkyo Technos Co., Ltd., Japan) at a flow rate of 300 nL/min. The mobile phases consisted of 0.1% formic acid in water (solvent A) and 0.1% formic acid in 80% acetonitrile (solvent B). The top 10 most intense precursor ions detected in the full MS scans were selected for fragmentation using data-dependent MS/MS analysis. MS/MS spectra were searched against the *Homo sapiens* Swiss-Prot database (version 2024/11/11) or the *Mus musculus* Swiss-Prot database (version 2022/06/14) using the Proteome Discoverer software (version 3.0) with the Sequest HT search engine. The search parameters were set as follows: maximum missed cleavages, 1; peptide length, 5–50 amino acids; precursor mass tolerance, 10 ppm; fragment mass tolerance, 0.02 Da. Carbamidomethylation of cysteine residues (+ 57.021 Da) was specified as static modification.

### Analysis of serum samples and clinical data from patients with sepsis

The clinical study was conducted in accordance with the study protocol approved by the Ethics Committees of Hokkaido University (Approval No. 022–0062) and Kumamoto University (Approval No. 2978). Serum samples were obtained from 58 patients with sepsis who were admitted to the Emergency and Critical Care Center of Hokkaido University Hospital between September 2019 and October 2021. The following variables were extracted and analyzed: age, sex, diagnosis, sequential organ failure assessment (SOFA) score, DIC score, and clinical outcomes at discharge.

### ELISA

Serum concentrations of cathelicidin antimicrobial peptide (Invitrogen, Waltham, MA, USA), lactotransferrin (Abcam, Cambridge, UK), neutrophil gelatinase-associated lipocalin (NGAL; R&D Systems, Minneapolis, MN, USA), and resistin (R&D Systems) were measured in sera from healthy volunteers and patients with sepsis using commercially available ELISA kits according to the manufacturers’ instructions. Serum histone H3 levels were measured using ELISA, as previously described [17]. Absorbance was measured at 450 nm using a SpectraMax iD3 microplate reader (Molecular Devices, San Jose, CA, USA).

### Statistical analysis

Statistical analyses were performed using GraphPad Prism (version 9; GraphPad Software) and R software (version 4.5.1). For LC–MS/MS data, the spectral abundance factor (SAF), calculated by normalizing the number of peptide–spectrum matches to protein length, was used as a semi-quantitative measure of protein abundance. Differences in SAF values between groups were analyzed using Linear Models for Microarray Data. Missing SAF values were imputed with half of the minimum observed value. Comparisons of ELISA measurements between subgroups were performed using the Mann–Whitney U test. Correlations between serum protein levels and circulating cell-free DNA concentrations among patients with sepsis were assessed using Spearman’s rank correlation coefficient. Receiver operating characteristic (ROC) curve analysis was performed, and the area under the curve (AUC) with 95% confidence intervals was calculated to assess the ability of the biomarkers to discriminate in-hospital mortality, as well as the presence or development of DIC and multiple organ failure by day 5. DIC status was retrospectively determined by recalculating the DIC score using the overt DIC criteria updated in 2025 [18]. Multiple organ failure was defined as the presence of organ failure [19] in three or more organ systems, based on a sensitivity analysis (Supplementary Fig. 1). *P *value < 0.05 was considered statistically significant.

## Results

### In vitro stimulation of neutrophils enables collection of NET-related supernatants

**Fig. 1:**
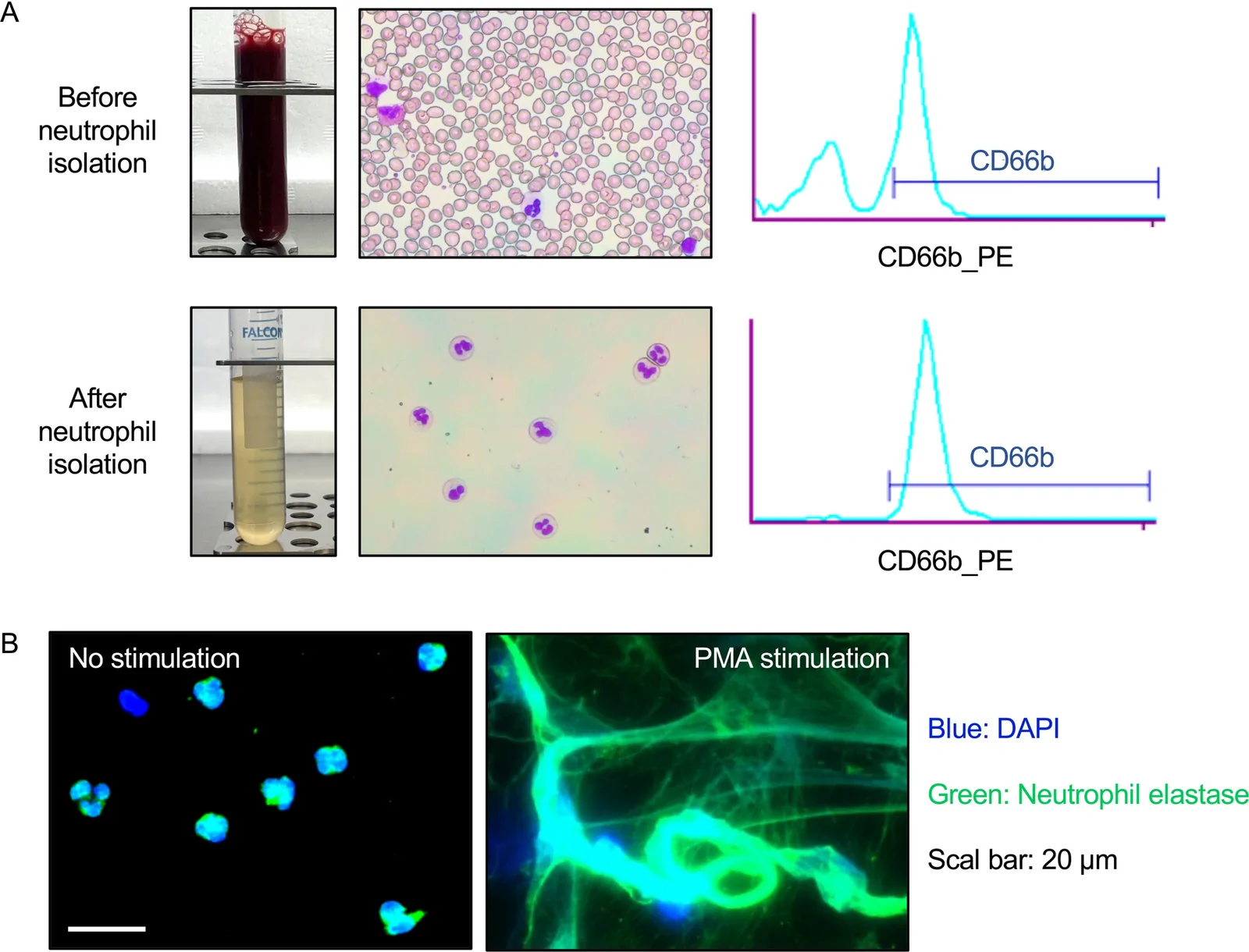
Isolation of human neutrophils and collection of culture supernatants after stimulation. **A** Representative images of whole blood (upper panels) and neutrophil-enriched fractions after isolation (lower panels). Macroscopic appearance (left), May–Grünwald–Giemsa-stained cytospin preparations (center), and flow cytometric analysis of CD66b-PE–positive cells (right) are shown. Following isolation, > 95% of the cells were CD66b-positive, confirming successful neutrophil enrichment. **B** Immunofluorescence images of unstimulated neutrophils (left) and neutrophils stimulated with 200 nM phorbol myristate acetate (PMA) for 4 h (right). DNA was stained with DAPI (blue), and neutrophil elastase is shown in green. Scale bars, 20 μm. PE: phycoerythrin; DAPI: 4′,6-diamidino-2-phenylindole

To identify soluble markers associated with NET release, we first established an in vitro system to induce NET formation and collected the proteins released into the culture supernatant. Neutrophils were isolated from the peripheral venous blood of four healthy donors (Fig. 1A). Isolated neutrophils were then cultured with or without PMA stimulation. In unstimulated cells, DNA was confined within multilobed nuclei, and NE was localized in the cytoplasm. In contrast, PMA-stimulated neutrophils exhibited a loss of cellular integrity, with both DNA and NE detected as extracellular web-like structures, consistent with NET formation (Fig. 1B). Culture supernatants from both conditions were collected and used for proteomic analysis.

### Proteomic profiling of neutrophil supernatants identifies proteins released during NET formation

**Fig. 2:**
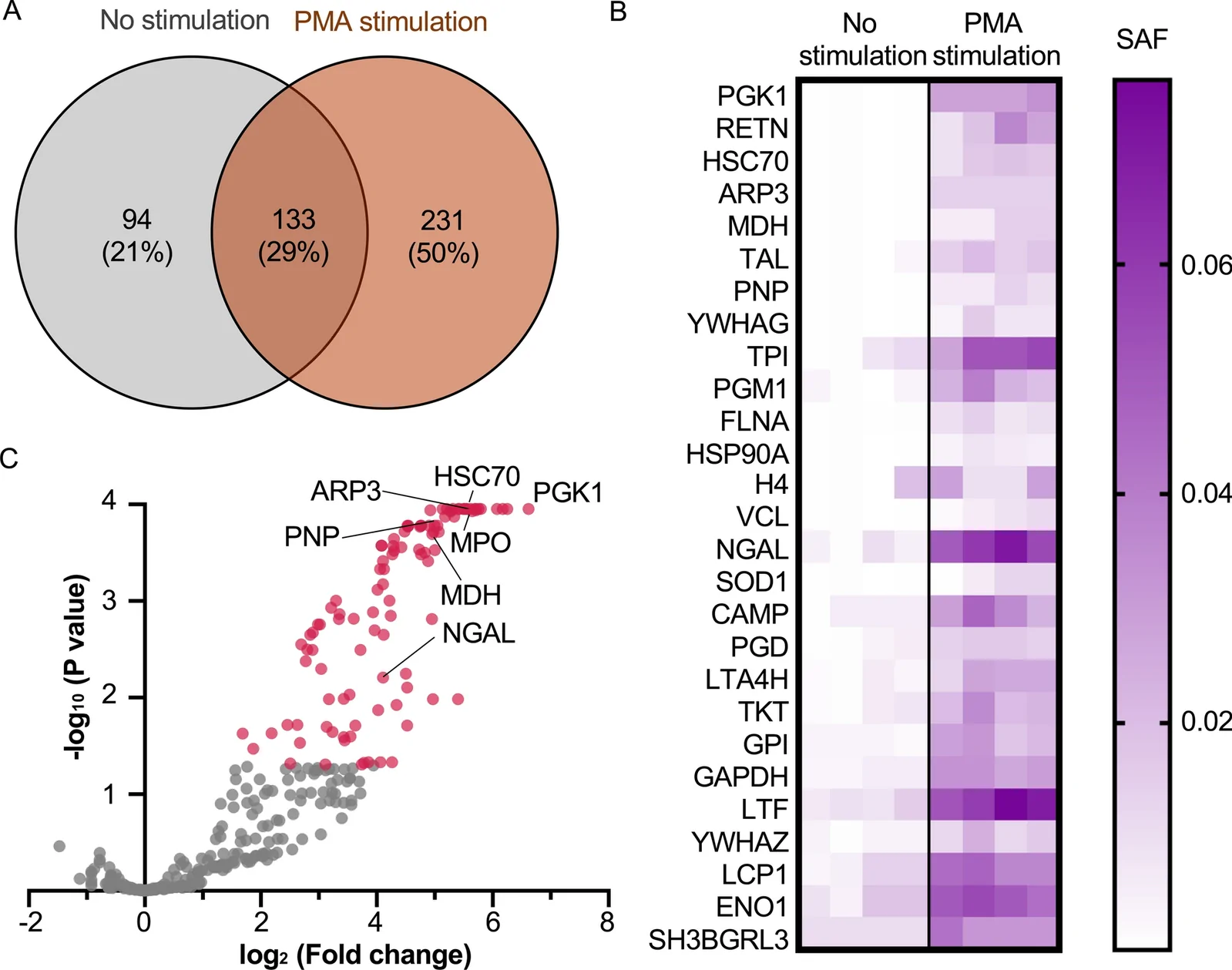
Proteomic profiling of neutrophil supernatants identifies proteins released during NET formation. **A** Venn diagram showing the overlap of proteins identified by LC–MS/MS in the supernatants of unstimulated neutrophils (*n* = 4) and PMA-stimulated neutrophils (*n* = 4). The numbers and percentages of identified proteins in each region are indicated. **B** Heatmap of SAF values for representative proteins increased in the supernatants following PMA stimulation. Higher intensity (purple) indicates a higher relative abundance. The full protein names corresponding to the abbreviations are provided in Supplementary Table 1. **C** Volcano plot based on the SAF values comparing protein abundance between unstimulated and PMA-stimulated supernatants. The x-axis represents the log fold change calculated from the mean SAF values, and the y-axis represents the negative logarithm of the *P* value. SAF: spectral abundance factor

To comprehensively characterize the proteins released during NET formation, LC–MS/MS was performed on the supernatants of unstimulated and PMA-stimulated neutrophils. As abundant albumin can interfere with protein detection, albumin was depleted prior to analysis (Supplementary Fig.  2 A). A total of 227 proteins were identified in the unstimulated group and 364 proteins in the PMA-stimulated group, of which 231 proteins were uniquely detected in the stimulated group (Fig. 2A). For semi-quantitative analysis, SAF values, defined as spectral counts normalized to protein length, were used. A total of 106 proteins exhibited significantly higher SAF values in the PMA-stimulated group than in the unstimulated group (Fig. 2B). These include proteins localized in specific granules (e.g., NGAL), members of the heat shock protein family (e.g., Hsc70), cytoskeletal proteins (e.g., actin-related protein 3), and metabolic enzymes (e.g., phosphoglycerate kinase-1, purine nucleoside phosphorylase, and malate dehydrogenase) (Fig. 2C). These results represent the first-stage screening for identifying proteins released into the extracellular milieu during NET formation.

### Proteomic profiling of serum identifies circulating proteins associated with sepsis in vivo

**Fig. 3:**
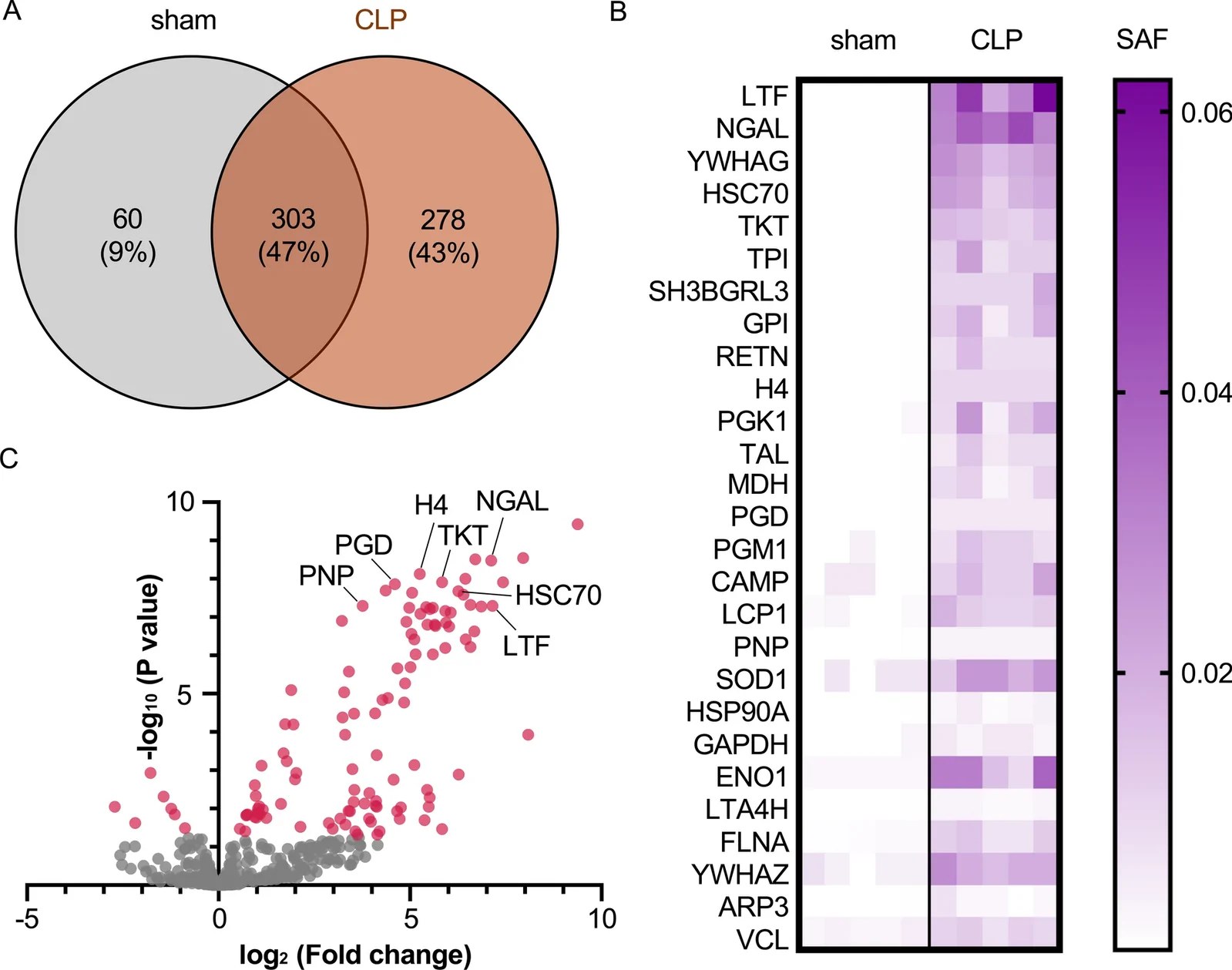
Proteomic profiling of serum identifies circulating proteins associated with sepsis in vivo*.* **A** Venn diagram showing the overlap of proteins identified by LC–MS/MS in the serum of mice subjected to cecal ligation and puncture (*n* = 5) and sham-operated controls (*n* = 5). The numbers and percentages of identified proteins in each region are indicated. **B** Heatmap of the SAF values for representative proteins increased in the serum following sepsis induction. Higher intensity (purple) indicates a higher relative abundance. **C** Volcano plot based on the SAF values comparing protein abundance between the sepsis and sham groups. The x-axis represents the log fold change calculated from the mean SAF values, and the y-axis represents the negative logarithm of the *P* value. SAF: spectral abundance factor

Next, we sought to identify proteins elevated in the circulation during sepsis. To minimize the inter-individual variability inherent to human clinical samples, a CLP mouse model was employed. Serum samples were subjected to LC–MS/MS following the depletion of abundant proteins, including albumin, IgG, and transferrin (Supplementary Fig. 2B). A total of 363 proteins were identified in sham-operated mice and 581 proteins in CLP mice, with 278 proteins uniquely detected in the CLP group (Fig. 3A). A total of 110 proteins showed significantly higher SAF values in the CLP group than in the sham group (Fig. 3B), including lactotransferrin, NGAL, histone H4, and 6-phosphogluconate dehydrogenase (Fig. 3C). This analysis constitutes the second-stage screening to identify circulating proteins that are elevated under septic conditions.

### Cell type-specific expression profiling prioritizes NET-related candidate proteins

**Fig. 4:**
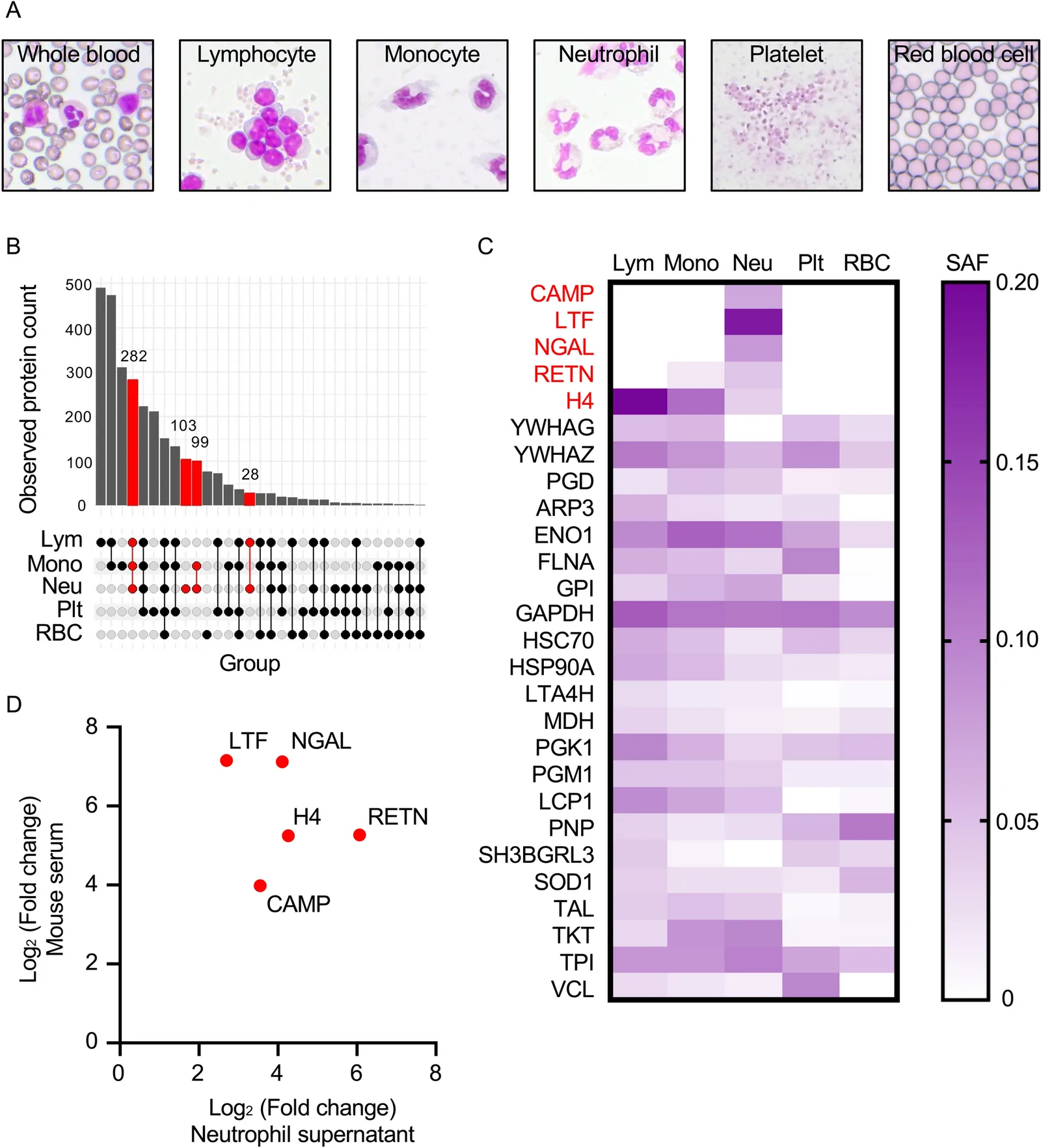
Cell type-specific expression profiling prioritizes NET-related candidate proteins. **A** Representative May–Grünwald–Giemsa-stained cytospin images of whole blood and isolated blood cell fractions, including lymphocytes, monocytes, neutrophils, platelets, and erythrocytes. **B** UpSet plot showing the number of proteins uniquely or commonly detected by LC–MS/MS in the cell lysates from different blood cell fractions. Proteins detected in leukocyte but not in platelet or erythrocyte fractions are highlighted in red. **C** Heatmap of SAF values in cell lysates from different blood cell fractions for 27 candidate proteins identified through integration of neutrophil supernatant and murine serum proteomic analyses. Color intensity (purple) indicates relative abundance. Proteins detected in leukocyte but not in platelet or erythrocyte fractions are highlighted in red. **D** Log fold changes calculated from mean SAF values in neutrophil supernatants and murine serum for the five selected candidate proteins following cell type-based filtering. These proteins were the final candidates prioritized for clinical validation. SAF: spectral abundance factor; Lym: lymphocyte; Mono: monocyte; Neu: neutrophil; Plt: platelet; RBC: red blood cell

Although the first- and second-stage screenings identified proteins released during NET formation and elevated in sepsis, this combined set still included a substantial number of broadly expressed proteins. To further refine the candidate NET-related soluble markers, we focused on proteins that are not ubiquitously expressed across blood cell types. To exclude broadly expressed proteins, LC–MS/MS analysis was performed on lysates from isolated neutrophils, monocytes, lymphocytes, platelets, and erythrocytes. The successful isolation of each cell type was confirmed by morphological examination (Fig. 4A). Among the detected proteins, 103 proteins were unique to neutrophils, 99 were shared between neutrophils and monocytes, 28 between neutrophils and lymphocytes, and 282 were common to neutrophils, monocytes, and lymphocytes (Fig. 4B). Among the 27 proteins identified in both the first- and second-stage screenings, the exclusion of proteins expressed in erythrocytes or platelets yielded five candidate molecules: cathelicidin antimicrobial peptide, lactotransferrin, NGAL, resistin, and histone H4 (Fig. 4C–D). These five proteins were selected for further analysis as candidate NET-related soluble markers of sepsis (Supplementary Fig. 3).

### Clinical validation identifies prognostic NET-related markers in sepsis

**Fig. 5:**
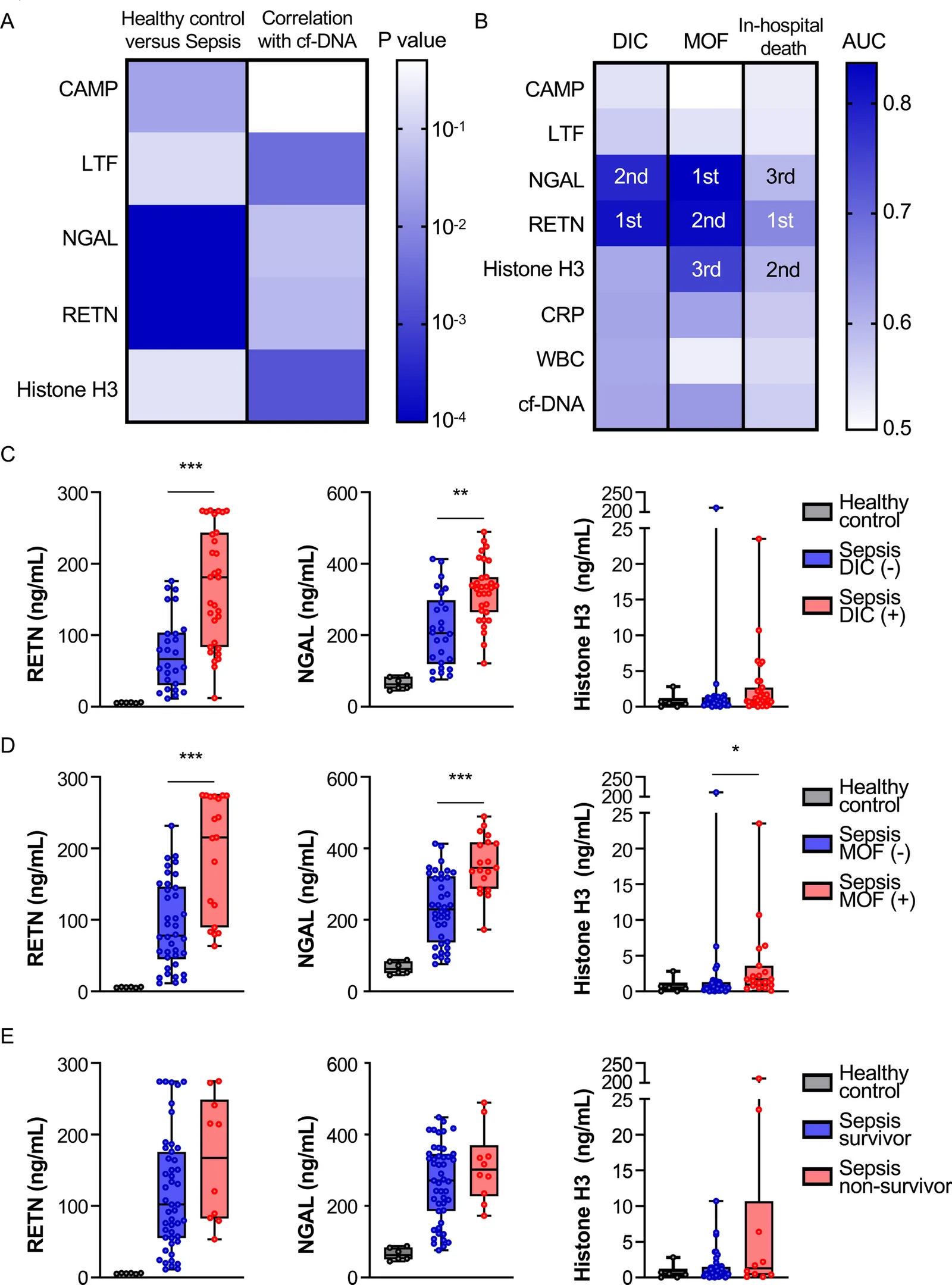
Clinical validation identifies prognostic NET-related markers in sepsis. **A** Heatmap of *P* values summarizing group comparisons and correlation analyses for candidate proteins measured in serum by enzyme-linked immunosorbent assay in healthy controls (*n* = 6) and patients with sepsis (*n* = 58) on the day of admission. *P* values for differences between groups were calculated using the Mann–Whitney U test, and P values for correlations with circulating cell-free DNA levels in patients with sepsis were obtained using Spearman’s rank correlation coefficients. **B** Heatmap of AUC values summarizing the discriminative performance of candidate proteins for disseminated intravascular coagulation (DIC), multiple organ failure, and in-hospital mortality in patients with sepsis, based on receiver operating characteristic analysis. **C** Box-and-whisker plots showing serum concentrations of resistin (left), NGAL (center), and histone H3 (right) in healthy controls, septic patients with or without DIC. **D** Serum concentrations of resistin (left), NGAL (center), and histone H3 (right) in healthy controls, septic patients with or without multiple organ failure. **E** Serum concentrations of resistin (left), NGAL (center), and histone H3 (right) in healthy controls, surviving or non-surviving septic patients. Boxes represent the interquartile range and median, and whiskers indicate the minimum and maximum values. *P < 0.01, **P < 0.001, ***P < 0.0001, Mann–Whitney U test AUC: area under the curve; DIC: disseminated intravascular coagulation; MOF: multiple organ failure

To determine whether the identified candidates were elevated in clinical settings and associated with disease severity, serum samples from six healthy volunteers and 58 patients with sepsis were analyzed. For histone H4, a suitable ELISA for serum samples was not available; therefore, a heterodimer-forming partner (histone H3), which is expected to exhibit similar behavior, was measured instead. Among the 58 patients, 10 (17%) died during hospitalization, 31 (53%) developed DIC by day 5, and 19 (33%) developed multiple organ failure by day 5 (Table 1). Detailed clinical and laboratory characteristics on day 1, including coagulation parameters and SOFA scores, are presented in Table 2. Compared to healthy controls, the serum levels of candidate proteins were elevated in patients with sepsis (Fig. 5A). To assess the association with NET formation, circulating cell-free DNA levels were used as a surrogate marker, given the difficulty in obtaining tissue specimens. All candidates, except cathelicidin antimicrobial peptide, showed positive correlations with cell-free DNA levels (Fig. 5A). ROC curve analysis demonstrated that the discriminative performance for DIC was highest for resistin (AUC 0.81, 95% CI 0.71–0.92), followed by NGAL (0.79, 95% CI 0.67–0.91) and histone H3 (0.62, 95% CI 0.47–0.76) (Supplementary Fig. 4, Fig. 5B and C). For multiple organ failure, NGAL showed the highest discriminative performance (AUC 0.84, 95% CI 0.73–0.94), followed by resistin (0.83, 95% CI 0.71–0.94) and histone H3 (0.75, 95% CI 0.62–0.89) (Supplementary Fig. 4, Fig. 5B and D). For in-hospital mortality, the discriminative performance was more modest, with resistin showing the highest AUC (0.66, 95% CI 0.48–0.84) (Supplementary Fig. 4, Fig. 5B and E). Notably, resistin and NGAL outperformed conventional clinical markers, including white blood cell count and C-reactive protein, in discriminating DIC, multiple organ failure, and mortality in patients with sepsis. These findings indicate that specific candidate proteins are significantly elevated in sepsis and may serve as useful markers for identifying severe disease.

**Table 1:** Baseline demographic and clinical characteristics

	Overall (*N* = 58)	Survivor (*N* = 48)	Non-survivor (*N* = 10)
Age (IQR)	77 (66–84)	77 (66–83)	78 (68–84)
Male	32 (55%)	28 (58%)	4 (40%)
Infection site			
Respiratory	18 (31%)	12 (25%)	6 (60%)
Abdominal	13 (22%)	11 (23%)	2 (20%)
Urinary tract	12 (21%)	11 (23%)	1 (10%)
Soft tissue	13 (22%)	12 (25%)	1 (10%)
Other	2 (3%)	2 (4%)	0 (0%)
DIC score (IQR)			
Day 1	3 (3–5)	3 (2–5)	4 (3–6)
Day 2	4 (3–5)	4 (3–5)	5 (3–6)
Day 3	4 (2–5)	3 (2–5)	6 (4–6)
Day 4	4 (2–5)	3 (2–4)	6 (4–6)
Day 5	4 (3–4)	3 (3–4)	6 (5–7)
DIC, day 1–5	31 (53%)	23 (48%)	8 (80%)
SOFA score (IQR)			
Day 1	9 (6–11)	8 (6–10)	12 (9–13)
Day 2	8 (5–11)	7 (4–10)	12 (10–12)
Day 3	6 (3–11)	5 (3–8)	12 (11–13)
Day 4	4 (2–9)	4 (2–6)	13 (5–15)
Day 5	3 (1–7)	3 (1–5)	8 (2–16)
SOFA subscores ≥ 3, day 1–5			
Respiratory	33 (57%)	24 (50%)	9 (90%)
Cardiovascular	42 (72%)	33 (69%)	9 (90%)
Renal	28 (48%)	19 (40%)	9 (90%)
Coagulation	20 (34%)	12 (25%)	8 (80%)
Liver	3 (5%)	2 (4%)	1 (10%)
Multiple organ failure, day 1–5	19 (33%)	11 (23%)	8 (80%)

**Table 2:** Laboratory and clinical characteristics on day 1

	Overall (*N* = 58)	Survivor (*N* = 48)	Non-survivor (*N* = 10)
Laboratory values (IQR)			
C-reactive protein, mg/dL	12.2 (5.3–2.3)	13.7 (4.9–25.5)	11.0 (8.1–12.9)
White blood cells,/µL	10.1 (5.1–18.6)	10.9 (5.3–18.1)	6.5 (3.8–19.7)
Cell free DNA, µg/mL	3.0 (2.3–6.8)	2.9 (2.3–5.6)	5.2 (2.1–15.3)
Platelet count, ×10^3^/μL	163 (77.3–247.5)	164.5 (82.3–229.5)	93.5 (68.3–256.8)
PT, sec	14.5 (13.3–19.9)	14.3 (13.3–17.8)	21.2 (13.8–41.0)
Fibrinogen, mg/dL	370.5 (298.0–590.5)	403.0 (316.3–601.8)	255.5 (167.0–389.8)
FDP, µg/mL	18.2 (8.6–34.0)	17.7 (8.4–32.0)	21.6 (9.6–42.5)
D-dimer, µg/mL	9.1 (3.7–20.9)	8.75 (3.4–19.7)	12.2 (4.2–21.1)
SOFA subscores (IQR)			
Respiratory	3 (2–3)	2 (2–3)	3 (3–4)
Cardiovascular	4 (0–4)	4 (0–4)	4 (4–4)
Renal	1 (1–3)	1 (1–3)	3 (2–4)
Coagulation	0 (0–2)	0 (0–2)	2 (0–2)
Liver	0 (0–1)	0 (0–1)	0 (0–0)

### Dynamic relocalization of resistin during NET formation

**Fig. 6:**
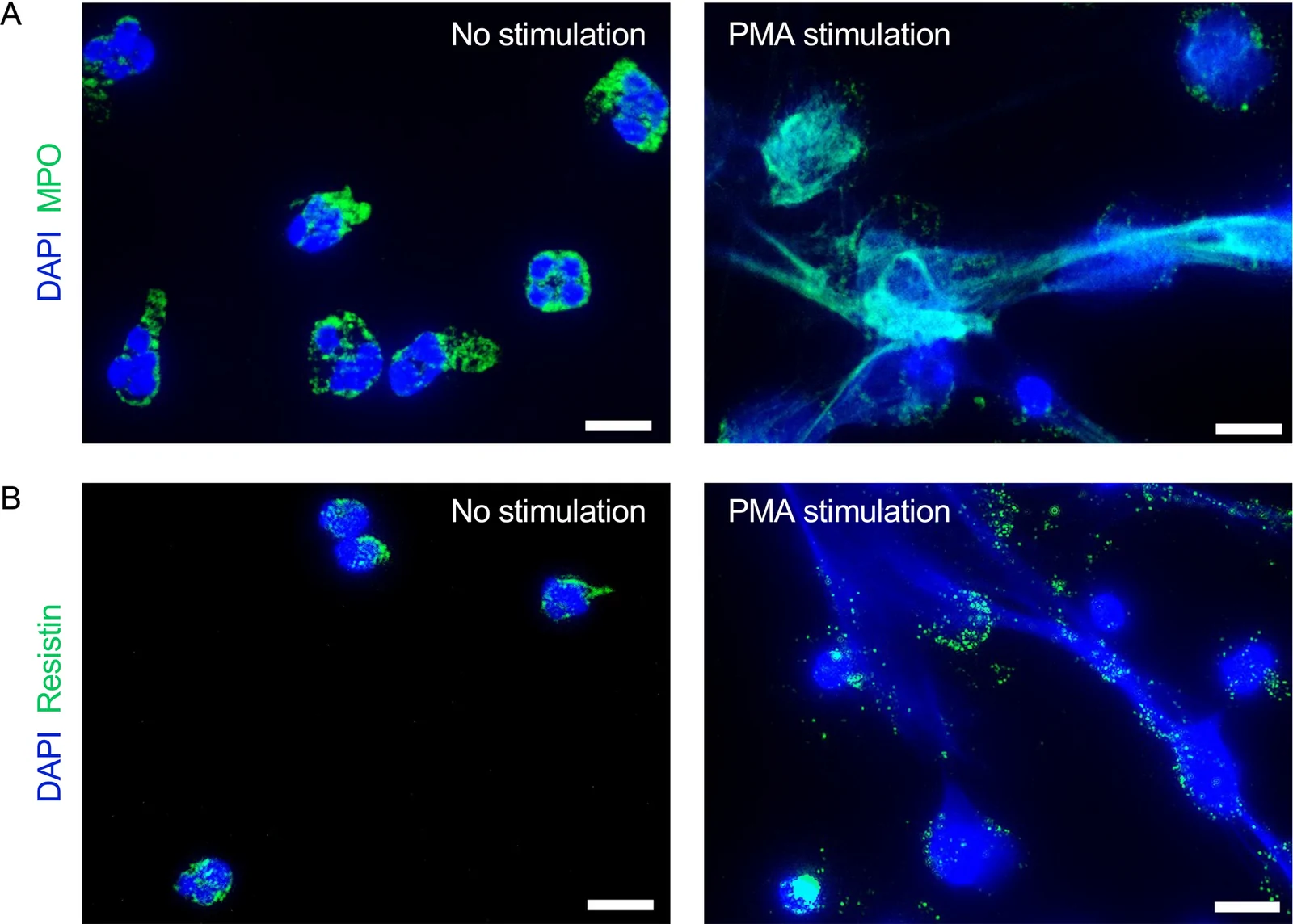
Dynamic relocalization of resistin during NET formation. **A** Immunofluorescence images of unstimulated neutrophils (left) and neutrophils stimulated with 200 nM PMA for 4 h (right). In unstimulated cells, DNA (blue) was confined to lobulated nuclei, and MPO (green) is localized within cytoplasmic granules. Upon stimulation, extracellular web-like structures containing DNA and myeloperoxidase were observed. **B** In unstimulated neutrophils (left), resistin (green) was localized within the cytoplasmic granules. Following PMA stimulation (right), extracellular DNA structures were observed, with only a minor fraction of resistin associated with these structures, while the majority was no longer detected within the cellular compartment. Scale bars, 10 μm. DAPI: 4′,6-diamidino-2-phenylindole; MPO: myeloperoxidase; PMA: phorbol myristate acetate

Finally, we examined the subcellular localization of resistin in neutrophils before and after NET formation. Immunofluorescence staining was performed alongside MPO, a key component of NET formation [20]. In unstimulated neutrophils, MPO was localized in the cytoplasmic granules and spatially separated from nuclear DNA. Following PMA stimulation, MPO and DNA co-localized in extracellular web-like structures, consistent with formation of NETs (Fig. 6A). Similarly, resistin was localized to the cytoplasm under the unstimulated condition. However, after PMA stimulation, resistin was largely absent from the cytoplasm of most neutrophils, while only a minimal signal was detected in extracellular DNA-associated structures (Fig. 6B). A similar localization pattern was observed for NGAL (Supplementary Fig. 5). These observations suggest that although resistin and NGAL are released during NET formation, they are not incorporated into the NET structure but are instead released in a soluble form, consistent with their potential as circulating biomarkers.

## Discussion

In this study, we combined in vitro proteomic profiling of neutrophil supernatants, in vivo proteomic analysis of serum from a murine sepsis model, and validation using clinical specimens to identify NET-related soluble markers. Through this stepwise and integrative approach, we demonstrated that resistin and NGAL are promising candidate biomarkers associated with NET formation in sepsis.

NGAL was originally described as a 25-kDa protein secreted by neutrophils upon PMA stimulation in 1993 [21]. In addition to neutrophils, NGAL is expressed in several non-hematopoietic cell types, including renal tubular epithelial cells, hepatocytes, and alveolar epithelial cells, and its expression is upregulated under inflammatory and ischemic conditions. Subsequently, its utility as a urinary biomarker for early ischemic acute kidney injury (AKI) was proposed [22], and numerous studies have demonstrated a close association between NGAL levels and AKI in both urine and blood samples [23, 24]. In our cohort, serum NGAL levels tended to increase with increasing renal SOFA subscores (Supplementary Fig. 6). These findings suggest that renal dysfunction may contribute to elevated NGAL levels, but may not fully account for the observed association between NGAL and disease severity. Higher plasma NGAL levels are associated with an increased risk of mortality and multiple organ failure [25]. NGAL has also been suggested to be a component of NETs [26]. In line with these observations, our results demonstrated that serum NGAL levels showed strong discriminative performance for multiple organ failure and DIC, supporting its potential utility as a prognostic biomarker of sepsis. However, its elevation in sepsis is unlikely to be specific to this process because NGAL can also be released from multiple cell types in response to systemic inflammation and organ injury. Further studies are needed to elucidate the mechanisms underlying NGAL elevation in sepsis, including the relative contribution of NET formation, and to validate its prognostic utility in prospective cohorts with sepsis.

Along with NGAL, our study identified resistin as a strong candidate NET-associated soluble marker. Resistin was originally discovered in adipocytes from diabetic mouse models and named based on its proposed role in insulin resistance [27]. However, subsequent studies have demonstrated that, in humans, resistin is predominantly expressed in immune cells, including neutrophils, monocytes, macrophages, and lymphocytes, rather than in adipocytes [28]. Functionally, resistin exerts direct antimicrobial effects by disrupting pathogen membranes and indirectly contributes to host defense by promoting immune cell recruitment, activation, inflammatory cytokine production, and NET formation [28]. Previous clinical studies have shown that resistin has a strong diagnostic performance for sepsis [29]. Moreover, higher serum resistin levels have been associated with increased disease severity, as reflected by SOFA scores, and poorer survival in specific subgroups of patients with sepsis [30]. These findings are consistent with our results, further supporting the potential of resistin as a prognostic biomarker for sepsis.

Beyond its role as a biomarker, resistin may also be a potential therapeutic target. While its antimicrobial activity is beneficial for host defense, recent studies suggest that resistin may exert cytotoxic effects on host tissues. For example, neutrophil-derived resistin has been shown to induce cardiomyocyte injury in the ischemic myocardium [31]. Although its role in sepsis remains incompletely defined, it is plausible that resistin released from activated neutrophils in the vasculature may damage endothelial cells, thereby promoting intravascular coagulation and contributing to organ dysfunction. Further studies are needed to elucidate the pathogenic versus protective roles of resistin in sepsis and to determine whether modulation of resistin could improve clinical outcomes.

This study has several limitations. Although our integrative approach identified proteins consistently detected in both in vitro NET-inducing conditions and in vivo sepsis models, we did not establish a causal or exclusive link between NET formation and increased resistin or NGAL levels. These proteins may reflect broader neutrophil activation or inflammatory responses that accompany, but are not specific to, NET release. Accordingly, our findings should be interpreted as identifying NET-associated, rather than NET-specific, soluble markers. Second, the use of SAF for semi-quantitative proteomic analysis may introduce bias due to differences in peptide digestion efficiency and inherent limitations of data-dependent acquisition. However, the final candidate molecules were quantitatively validated using ELISA in clinical samples, which supports the robustness of our observations. Third, because different species were used in the first (human neutrophils) and second (murine serum) screening stages, species-specific differences may have led to the omission of certain candidate proteins. Fourth, the specificity of resistin and NGAL for sepsis remains to be fully established, as both proteins have been reported to be elevated in other conditions, including AKI, chronic inflammatory diseases, metabolic disorders, and malignancies [28, 32]. Finally, the clinical validation was retrospective and conducted in a relatively small cohort; therefore, prospective studies in larger populations are required to confirm these findings.

## Conclusion

Using an integrative and unbiased proteomic strategy, we identified a set of soluble proteins released from neutrophils in association with NET formation. Among these, resistin and NGAL demonstrated strong associations with DIC and multiple organ failure in patients with sepsis. These findings provide a basis for the development of NET-related soluble markers as tools for risk stratification in patients with sepsis.

## Supplementary Information

Supplementary material 1

Supplementary material 2

## Data Availability

The datasets generated and/or analyzed during the current study are available from the corresponding author on reasonable request.

## References

[1] Singer M, Deutschman CS, Seymour CW, Shankar-Hari M, Annane D, Bauer M, et al. The Third International Consensus Definitions for Sepsis and Septic Shock (Sepsis-3). JAMA. 2016;315(8):801–10. 10.1001/jama.2016.0287 PMC4968574 26903338

[2] World Health Organization. Organization WH. Sepsis: Fact sheet. https://www.who.int/news-room/fact-sheets/detail/sepsis? Accessed 18 May 2026.

[3] Rudd KE, Johnson SC, Agesa KM, Shackelford KA, Tsoi D, Kievlan DR, et al. Global, regional, and national sepsis incidence and mortality, 1990–2017: analysis for the Global Burden of Disease Study. Lancet. 1990;395(10219):200–11. 10.1016/S0140-6736(19)32989-7 PMC6970225 31954465

[4] Arefian H, Heublein S, Scherag A, Brunkhorst FM, Younis MZ, Moerer O, et al. Hospital-related cost of sepsis: a systematic review. J Infect. 2017;74(2):107–17. 10.1016/j.jinf.2016.11.006 27884733

[5] Othman A, Sekheri M, Filep JG. Roles of neutrophil granule proteins in orchestrating inflammation and immunity. FEBS J. 2022;289(14):3932–53. 10.1111/febs.15803 PMC9546106 33683814

[6] Gierlikowska B, Stachura A, Gierlikowski W, Demkow U. The impact of cytokines on neutrophils’ phagocytosis and NET formation during sepsis-a review. Int J Mol Sci. 2022. 10.3390/ijms23095076. PMC9101385 35563475

[7] Brinkmann V, Reichard U, Goosmann C, Fauler B, Uhlemann Y, Weiss DS, et al. Neutrophil extracellular traps kill bacteria. Science. 2004;303(5663):1532–5. 10.1126/science.1092385 15001782

[8] Wang Y, Du C, Zhang Y, Zhu L. Composition and function of neutrophil extracellular traps. Biomolecules. 2024. 10.3390/biom14040416. PMC11048602 38672433

[9] Parker H, Albrett AM, Kettle AJ, Winterbourn CC. Myeloperoxidase associated with neutrophil extracellular traps is active and mediates bacterial killing in the presence of hydrogen peroxide. J Leukoc Biol. 2012;91(3):369–76. 10.1189/jlb.0711387 22131345

[10] Liao F, Fan J, Wang R, Xu Z, Li Q, Bi W, et al. Neutrophil extracellular traps in sepsis: trade-off between pros and cons. Burns Trauma. 2025;13:tkaf046. 10.1093/burnst/tkaf046 PMC12403064 40904580

[11] Silvestre-Roig C, Braster Q, Wichapong K, Lee EY, Teulon JM, Berrebeh N, et al. Externalized histone H4 orchestrates chronic inflammation by inducing lytic cell death. Nature. 2019;569(7755):236–40. 10.1038/s41586-019-1167-6 PMC6716525 31043745

[12] Ito T, Kakuuchi M, Maruyama I. Endotheliopathy in septic conditions: mechanistic insight into intravascular coagulation. Crit Care. 2021;25(1):95. 10.1186/s13054-021-03524-6 PMC7938685 33685461

[13] von Bruhl ML, Stark K, Steinhart A, Chandraratne S, Konrad I, Lorenz M, et al. Monocytes, neutrophils, and platelets cooperate to initiate and propagate venous thrombosis in mice in vivo. J Exp Med. 2012;209(4):819–35. 10.1084/jem.20112322 PMC3328366 22451716

[14] Iba T, Helms J, Connors JM, Levy JH. The pathophysiology, diagnosis, and management of sepsis-associated disseminated intravascular coagulation. J Intensive Care. 2023;11(1):24. 10.1186/s40560-023-00672-5 PMC10202753 37221630

[15] Stoimenou M, Tzoros G, Skendros P, Chrysanthopoulou A. Methods for the assessment of NET formation: from neutrophil biology to translational research. Int J Mol Sci. 2022. 10.3390/ijms232415823. PMC9781911 36555464

[16] Rittirsch D, Huber-Lang MS, Flierl MA, Ward PA. Immunodesign of experimental sepsis by cecal ligation and puncture. Nat Protoc. 2009;4(1):31–6. 10.1038/nprot.2008.214 PMC2754226 19131954

[17] Ito T, Nakahara M, Masuda Y, Ono S, Yamada S, Ishikura H, et al. Circulating histone H3 levels are increased in septic mice in a neutrophil-dependent manner: preclinical evaluation of a novel sandwich ELISA for histone H3. J Intensive Care. 2018;6:79. 10.1186/s40560-018-0348-y PMC6260889 30505450

[18] Iba T, Levy JH, Maier CL, Helms J, Umemura Y, Moore H, et al. Updated definition and scoring of disseminated intravascular coagulation in 2025: communication from the ISTH SSC Subcommittee on Disseminated Intravascular Coagulation. J Thromb Haemost. 2025;23(7):2356–62. 10.1016/j.jtha.2025.03.038 40216223

[19] Moreno R, Vincent JL, Matos R, Mendonca A, Cantraine F, Thijs L, et al. The use of maximum SOFA score to quantify organ dysfunction/failure in intensive care. Results of a prospective, multicentre study. Working Group on Sepsis related Problems of the ESICM. Intensive Care Med. 1999;25(7):686–96. 10.1007/s001340050931 10470572

[20] Burn GL, Raisch T, Tacke S, Winkler M, Prumbaum D, Thee S, et al. Myeloperoxidase transforms chromatin into neutrophil extracellular traps. Nature. 2025;647(8090):747–56. 10.1038/s41586-025-09523-9 PMC12629992 40963017

[21] Kjeldsen L, Johnsen AH, Sengelov H, Borregaard N. Isolation and primary structure of NGAL, a novel protein associated with human neutrophil gelatinase. J Biol Chem. 1993;268(14):10425–32. 7683678

[22] Mishra J, Ma Q, Prada A, Mitsnefes M, Zahedi K, Yang J, et al. Identification of neutrophil gelatinase-associated lipocalin as a novel early urinary biomarker for ischemic renal injury. J Am Soc Nephrol. 2003;14(10):2534–43. 10.1097/01.asn.0000088027.54400.c6 14514731

[23] Mishra J, Dent C, Tarabishi R, Mitsnefes MM, Ma Q, Kelly C, et al. Neutrophil gelatinase-associated lipocalin (NGAL) as a biomarker for acute renal injury after cardiac surgery. Lancet. 2005;365(9466):1231–8. 10.1016/S0140-6736(05)74811-X 15811456

[24] Haase M, Bellomo R, Devarajan P, Schlattmann P, Haase-Fielitz A, Grp NMAI. Accuracy of neutrophil gelatinase-associated lipocalin (NGAL) in diagnosis and prognosis in acute kidney injury: a systematic review and meta-analysis. Am J Kidney Dis. 2009;54(6):1012–24. 10.1053/j.ajkd.2009.07.020 19850388

[25] Wang B, Chen G, Zhang J, Xue J, Cao Y, Wu Y. Increased neutrophil gelatinase-associated lipocalin is associated with mortality and multiple organ dysfunction syndrome in severe sepsis and septic shock. Shock. 2015;44(3):234–8. 10.1097/SHK.0000000000000408 26009825

[26] Li H, Feng D, Cai Y, Liu Y, Xu M, Xiang X, et al. Hepatocytes and neutrophils cooperatively suppress bacterial infection by differentially regulating lipocalin-2 and neutrophil extracellular traps. Hepatology. 2018;68(4):1604–20. 10.1002/hep.29919 PMC6173649 29633303

[27] Steppan CM, Bailey ST, Bhat S, Brown EJ, Banerjee RR, Wright CM, et al. The hormone resistin links obesity to diabetes. Nature. 2001;409(6818):307–12. 10.1038/35053000 11201732

[28] Li Y, Yang Q, Cai D, Guo H, Fang J, Cui H, et al. Resistin, a novel host defense peptide of innate immunity. Front Immunol. 2021;12:699807. 10.3389/fimmu.2021.699807 PMC8253364 34220862

[29] Lan Y, Guo W, Chen W, Chen M, Li S. Resistin as a potential diagnostic biomarker for sepsis: insights from DIA and ELISA analyses. Clin Proteomics. 2024;21(1):46. 10.1186/s12014-024-09498-1 PMC11218185 38951753

[30] Chen J, Liu Z, Zhou F, Sun Y, Jiang Z, Zhao P. Assessment of S100A8/A9 and resistin as predictive biomarkers for mortality in critically ill patients with sepsis. Front Cell Infect Microbiol. 2025;15:1555307. 10.3389/fcimb.2025.1555307 PMC12188459 40568710

[31] Kumowski N, Pabel S, Grune J, Momin N, Ninh VK, Stengel L, et al. Resistin-like molecule gamma attacks cardiomyocyte membranes and promotes ventricular tachycardia. Science. 2025;389(6764):1043–8. 10.1126/science.adp7361 PMC12805919 40906843

[32] Bokarewa M, Nagaev I, Dahlberg L, Smith U, Tarkowski A. Resistin, an adipokine with potent proinflammatory properties. J Immunol. 2005;174(9):5789–95. 10.4049/jimmunol.174.9.5789 15843582

