# Supplementary material for: Proteomic identification of resistin as a NET-associated neutrophil activity marker in sepsis
Source: J Intensive Care. 2026 Oct 13;14:111. doi: 10.1186/s40560-026-00954-8 (PMC13660192; doi:10.1186/s40560-026-00954-8)
Supplement: Supplementary file 1 [file 40560_2026_954_MOESM1_ESM.pdf]

Supplementary Figure 1

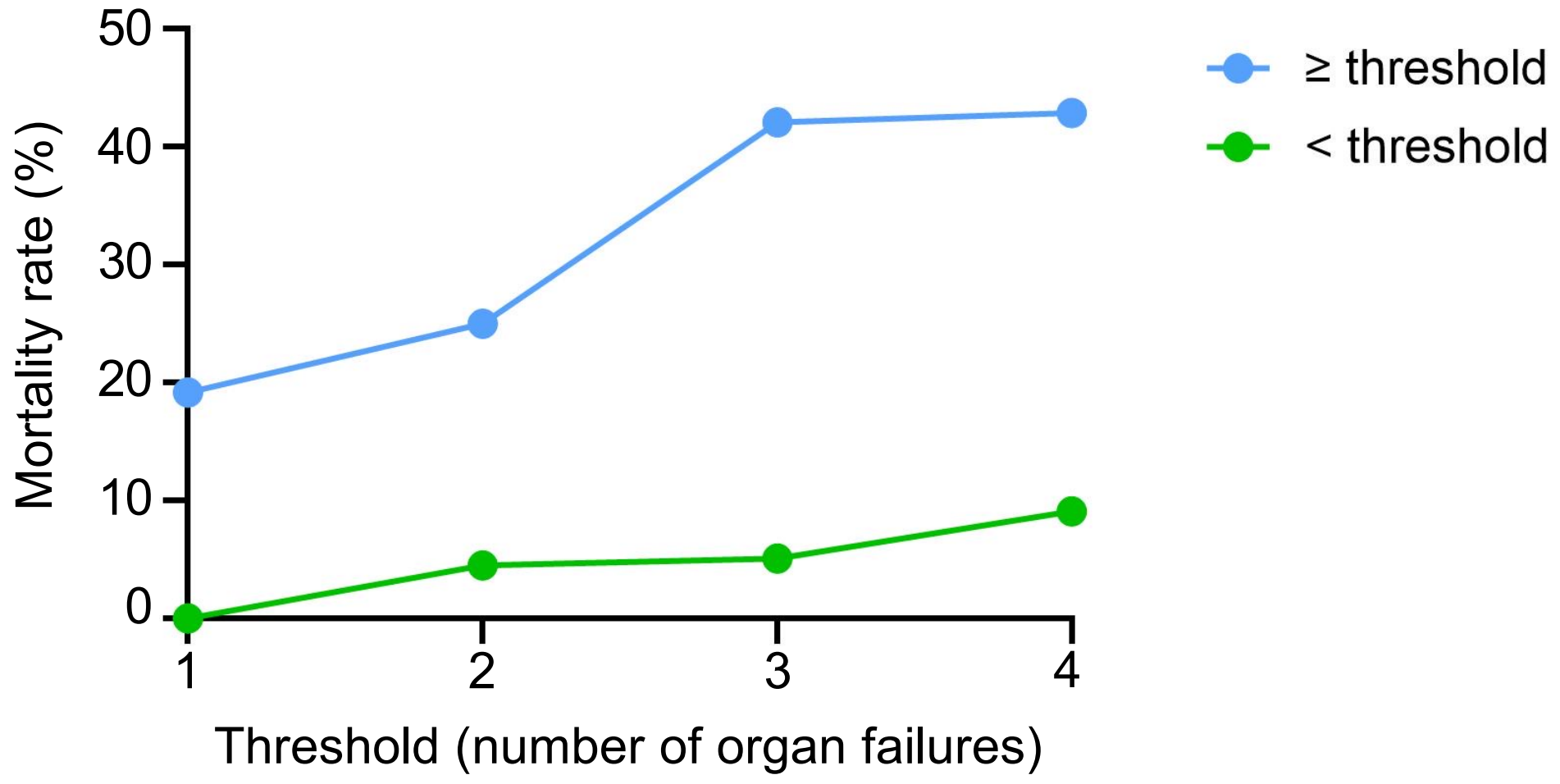


**Supplementary Figure 1. Sensitivity analysis of in-hospital mortality according to the number of organ failures.** In-hospital mortality was compared using thresholds of ≥1, ≥2, ≥3, and ≥4 organ failures. Organ failure was defined as a SOFA subscore ≥3 for each organ system. The greatest difference in mortality was observed at a threshold of three organ failures.

Supplementary Figure 2

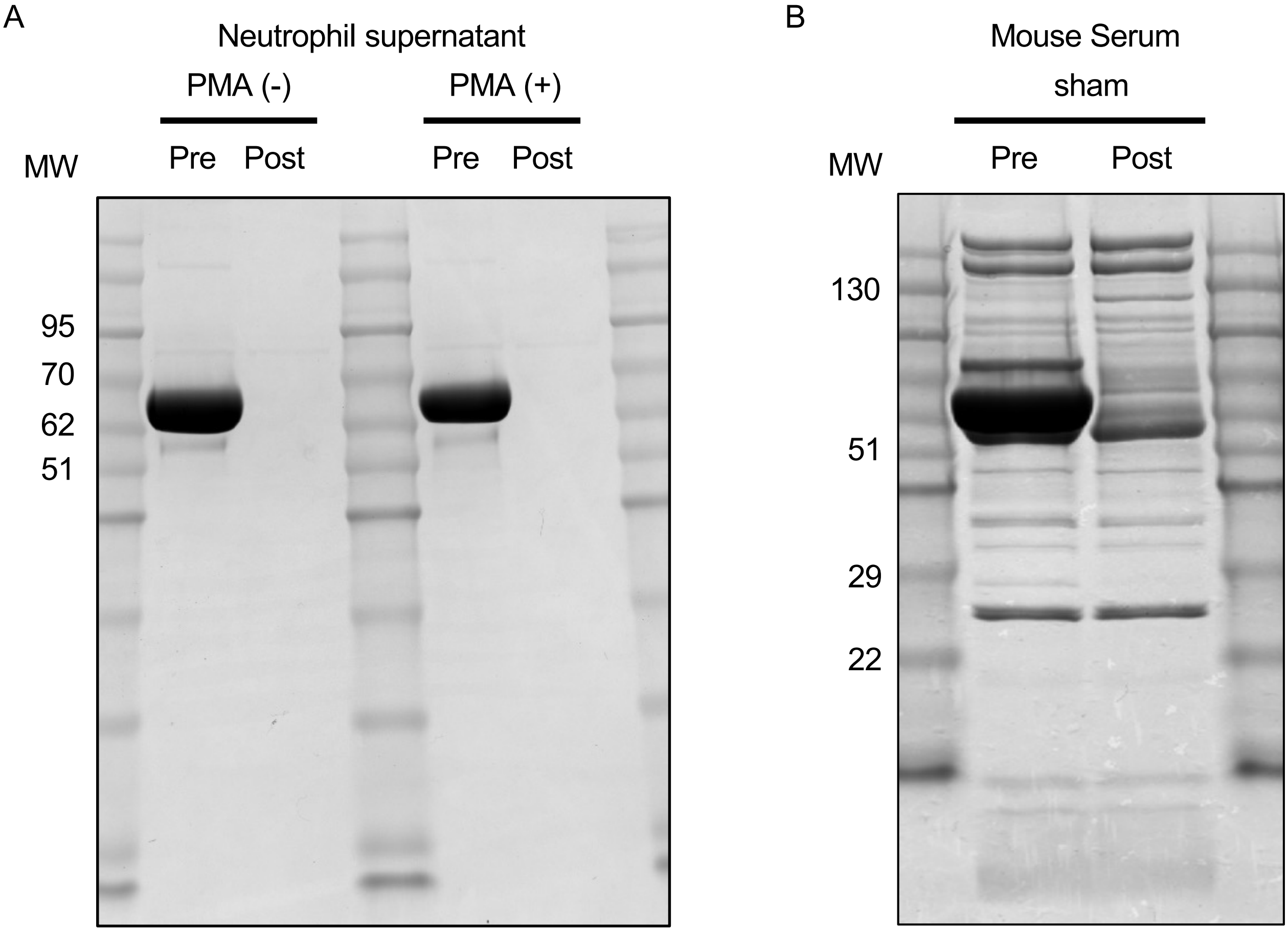


**Supplementary Figure 2. Albumin depletion from neutrophil culture supernatants and murine serum.** (A) Neutrophil culture supernatants before (pre) and after (post) albumin depletion were separated by SDS-PAGE and visualized by Coomassie Brilliant Blue staining. (B) Murine serum before (pre) and after (post) depletion of albumin, IgG, and transferrin were separated by SDS-PAGE and analyzed by Coomassie Brilliant Blue staining.

Supplementary Figure 3

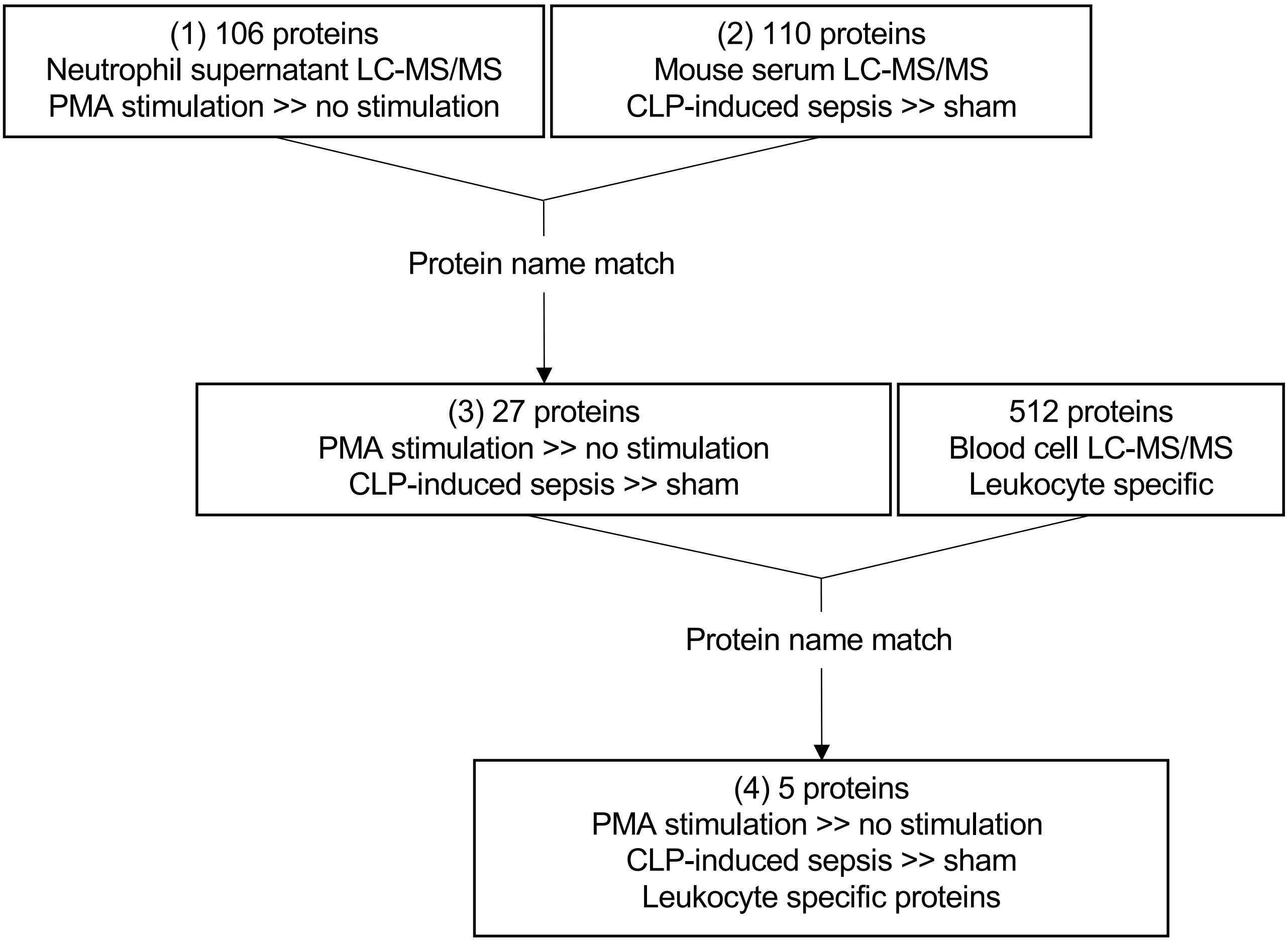


**Supplementary Figure 3. Flowchart for selection of candidate NET-associated soluble markers.** (1) A total of 106 proteins were identified as increased in PMA-stimulated neutrophil culture supernatants. (2) A total of 110 proteins were identified as increased in serum from the cecal ligation and puncture (CLP) mouse model. (3) Twenty-seven proteins were commonly identified in both datasets. (4) Of these, proteins detected in erythrocytes or platelets were excluded due to lack of leukocyte specificity, resulting in five final candidate NET-associated soluble markers.

Supplementary Figure 4

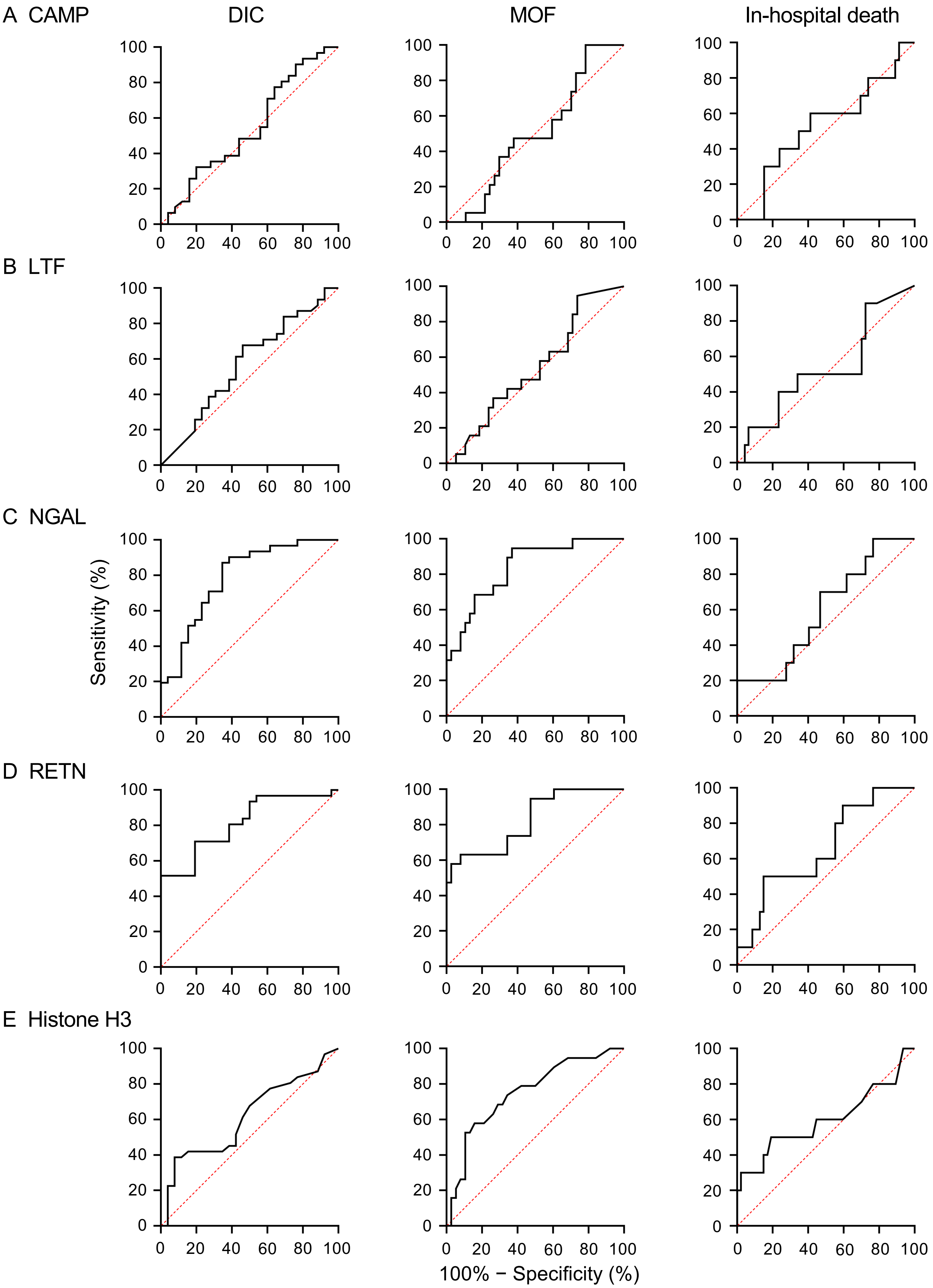


**Supplementary Figure 4. Discriminative performance of the candidate proteins for clinical outcomes in patients with sepsis.** ROC curves of (A) CAMP, (B) LTF, (C) NGAL, (D) RETN, and (E) Histone H3 for disseminated intravascular coagulation (DIC), multiple organ failure (MOF), and in-hospital mortality are shown. The diagonal dashed lines indicate the reference line representing no discrimination (AUC = 0.5).

Supplementary Figure 5

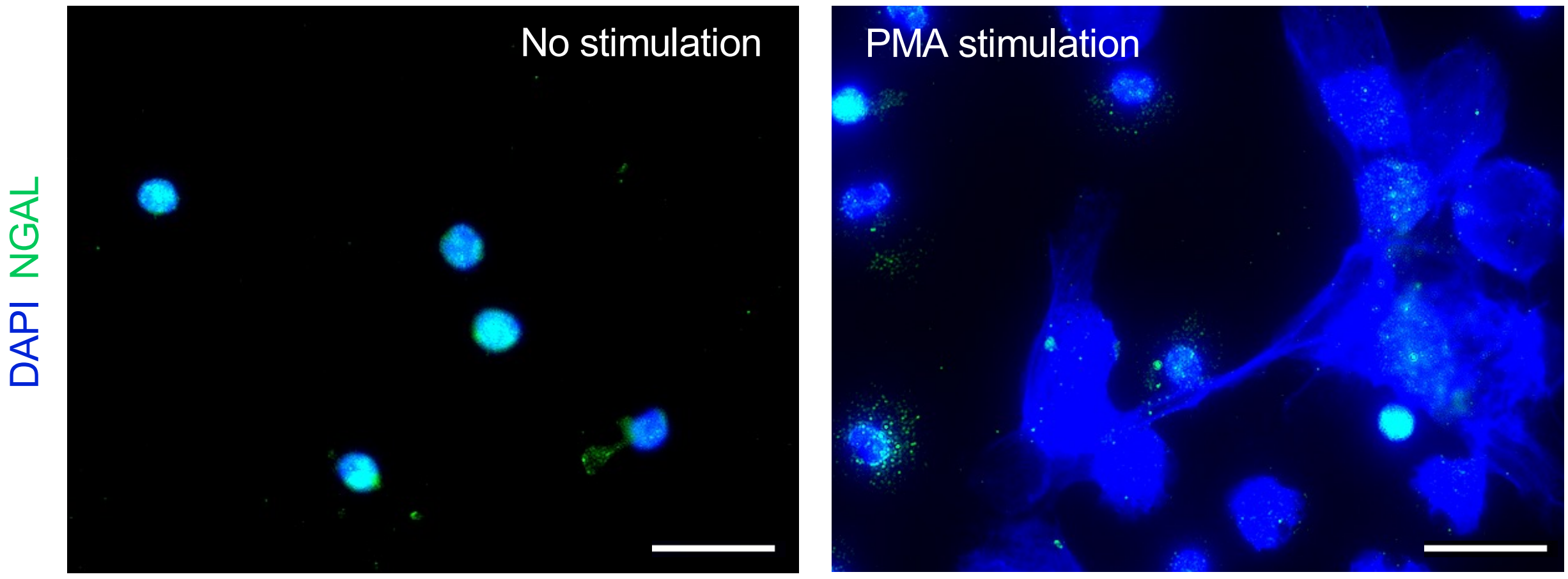


**Supplementary Figure 5. Dynamic relocalization of NGAL during NET formation.** In unstimulated neutrophils (left), NGAL (green) was localized within cytoplasmic granules. Following PMA stimulation (right), extracellular DNA structures were observed, with only a minor fraction of NGAL associated with these structures, while the majority was no longer detected within the cellular compartment. Scale bars, 20 μm.

NGAL, neutrophil gelatinase-associated lipocalin; DAPI, 4′,6-diamidino-2-phenylindole; PMA, phorbol myristate acetate.

Supplementary Figure 6

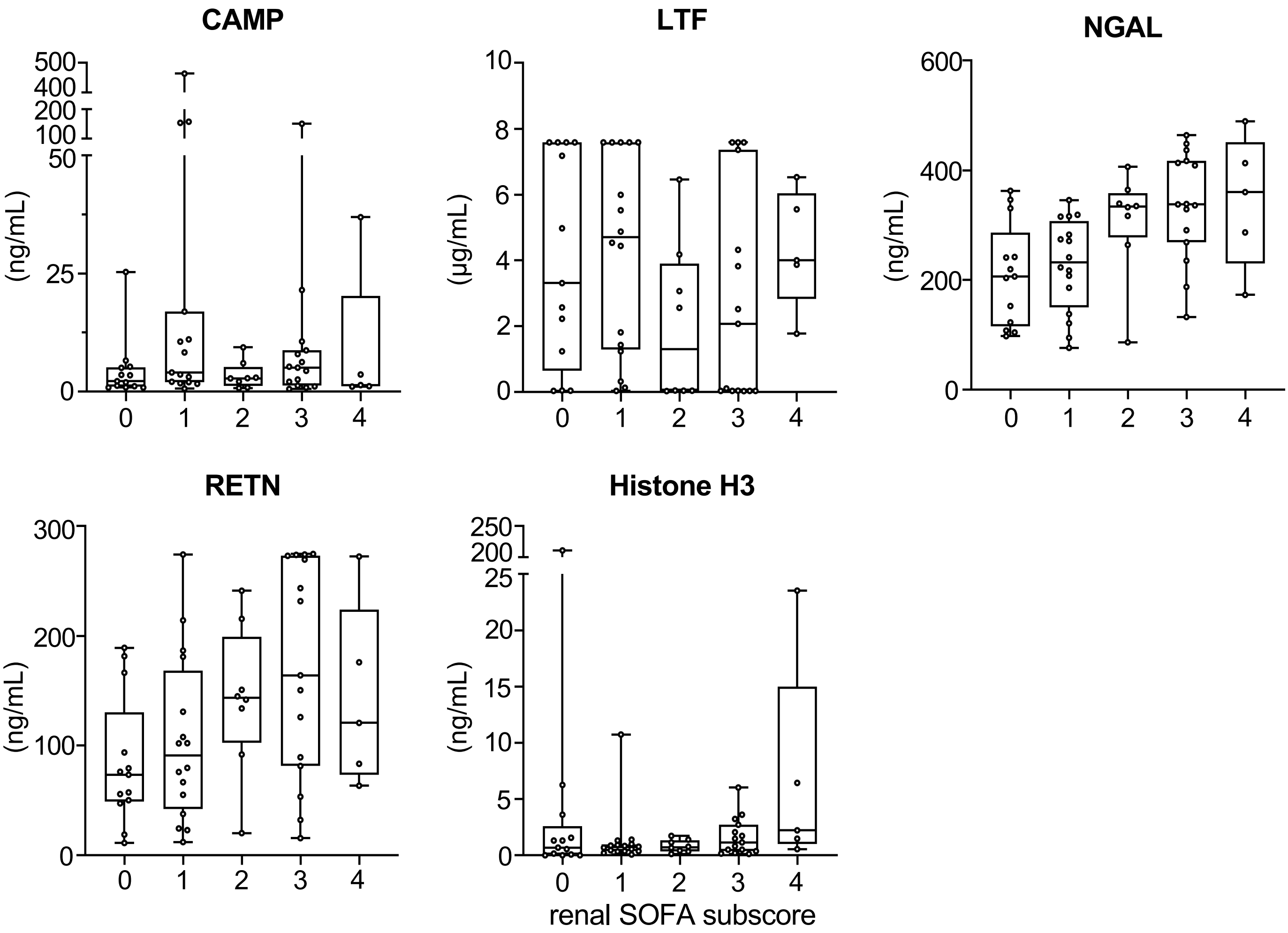


**Supplementary Figure 6. Serum concentrations of the candidate biomarkers according to the renal SOFA subscore on day 1.** Box-and-whisker plots showing serum concentrations of CAMP, LTF, NGAL, RETN, and Histone H3 across renal SOFA subscores (0–4) on day 1.

CAMP, cathelicidin antimicrobial peptide; LTF, lactotransferrin; NGAL, neutrophil gelatinase-associated lipocalin; RETN, resistin.
