# Supplementary material for: Proteomic identification of resistin as a NET-associated neutrophil activity marker in sepsis
Source: J Intensive Care. 2026 Oct 13;14:111. doi: 10.1186/s40560-026-00954-8 (PMC13660192; doi:10.1186/s40560-026-00954-8)
Supplement: Supplementary file 2 [file 40560_2026_954_MOESM2_ESM.docx]

Supplementary Table 1. Abbreviations of identified proteins

| Abbreviation | Full name |
| --- | --- |
| ARP3 | Actin-related protein 3 |
| CAMP | Cathelicidin antimicrobial peptide |
| ENO1 | Alpha-enolase |
| FLNA | Filamin-A |
| GAPDH | Glyceraldehyde-3-phosphate dehydrogenase |
| GPI | Glucose-6-phosphate isomerase |
| H4 | Histone H4 |
| HSC70 | Heat shock cognate 71 kDa protein |
| HSP90A | Heat shock protein HSP 90-alpha |
| LCP1 | Plastin-2 |
| LTA4H | Leukotriene A-4 hydrolase |
| LTF | Lactotransferrin |
| MDH | Malate dehydrogenase, cytoplasmic |
| NGAL | Neutrophil gelatinase-associated lipocalin |
| PGD | 6-phosphogluconate dehydrogenase, decarboxylating |
| PGK1 | Phosphoglycerate kinase 1 |
| PGM1 | Phosphoglycerate mutase 1 |
| PNP | Purine nucleoside phosphorylase |
| RETN | Resistin |
| SH3BGRL3 | SH3 domain-binding glutamic acid-rich-like protein 3 |
| SOD1 | Superoxide dismutase [Cu-Zn] |
| TAL | Transaldolase |
| TKT | Transketolase |
| TPI | Triosephosphate isomerase |
| VCL | Vinculin |
| YWHAG | 14-3-3 protein gamma |
| YWHAZ | 14-3-3 protein zeta/delta |
